# The Effects of Massage Therapy on Sport and Exercise Performance: A Systematic Review

**DOI:** 10.3390/sports11060110

**Published:** 2023-05-29

**Authors:** Miloš Dakić, Lazar Toskić, Vladimir Ilić, Saša Đurić, Milivoj Dopsaj, Jožef Šimenko

**Affiliations:** 1 Faculty of Sport and Physical Education, University of Belgrade, 11030 Belgrade, Serbia; miloshdakic@gmail.com (M.D.); vladimir.ilic@fsfv.bg.ac.rs (V.I.); milivoj.dopsaj@fsfv.bg.ac.rs (M.D.); 2 Faculty of Sport and Physical Education, University of Priština in Kosovska Mitrovica, 38218 Leposavić, Serbia; 3 Faculty of Sport, University “Union–Nikola Tesla”, 11070 Belgrade, Serbia; 4 Liberal Arts Department, American University of the Middle East, Egaila 54200, Kuwait; sasa.djuric@aum.edu.kw; 5 School of Life and Medical Sciences, University of Hertfordshire, Hatfield AL10 9AB, UK

**Keywords:** recovery, motor abilities, neurophysiology, psychology, performance

## Abstract

Background: A massage is a tool that is frequently used in sports and exercise in general for recovery and increased performance. In this review paper, we aimed to search and systemize current literature findings relating to massages’ effects on sports and exercise performance concerning its effects on motor abilities and neurophysiological and psychological mechanisms. Methods: The review has been written following the PRISMA (Preferred Reporting Items for Systematic Reviews and Meta-analysis) guidelines. One hundred and fourteen articles were included in this review. Results: The data revealed that massages, in general, do not affect motor abilities, except flexibility. However, several studies demonstrated that positive muscle force and strength changed 48 h after the massage was given. Concerning neurophysiological parameters, the massage did not change blood lactate clearance, muscle blood flow, muscle temperature, or activation. However, many studies indicate pain reduction and delayed onset muscle soreness, which are probably correlated with the reduction of the level of creatine kinase enzyme and psychological mechanisms. In addition, the massage treatment led to a decrease in depression, stress, anxiety, and the perception of fatigue and an increase in mood, relaxation, and the perception of recovery. Conclusion: The direct usage of massages just for gaining results in sport and exercise performance seems questionable. However, it is indirectly connected to performance as an important tool when an athlete should stay focused and relaxed during competition or training and recover after them.

## 1. Introduction

Today’s regime of competitive sport is very intensive and often causes athletes to become fatigued. If the organism has not recovered enough between strenuous activities, it could eventually lead to overtraining or an injury. It is also necessary for recovery to be as fast and efficient as possible because, in real field situations, during breaks, an athlete has a couple of hours or sometimes only a few minutes to prepare for the next game/match. The difference between winning and losing is often the capability to optimally maintain muscle work despite fatigue. A sports massage represents a tool that is frequently used in sports for that purpose, to recover and prepare an athlete for the following match [1,2,3]. However, it has been highlighted that sports massage is time-consuming and expensive [3]. Therefore, the best and most efficient practices should be explored, which are also supported by evidence. 

A massage is generally defined as the mechanical manipulation of soft tissues using rhythmically applied moves and pressures with the purpose of enhancing health and well-being [1,2]. Besides manual massages, many other forms of massages are applied in sports. These are the vibro-massage, hydro-massage, acupressure massage, rolling massage (using myofascial release techniques), and massages with a foam roller (FR), which is the most used instrument in the sport and fitness industry today [4]. The literature frequently reports that massage therapy positively affects physiological, neurological, psychological, and biomechanical mechanisms [5]. Still, there have been many adverse results when researchers tried to test those hypotheses, as shown in the following sections of the manuscript. Despite this, the massage is the most often used medical treatment in sports competitions. For example, Galloway and Watt [2] and Ernst [6] observed athletes in Great Britain between 1987 and 1998 and showed that massage therapies had 45% more involvement than all other medical treatments did at big sports events. Additionally, the sports massage has been reported to generate a multi-million-GBP industry of professional therapists and massage accessories [3]. 

So far, several review studies have dealt with the effects of massage therapy on various properties of interest for sport and exercise. However, most of these studies were not conducted recently; so, the latest findings could not be included [5,7,8,9,10,11,12], and some of them were focused on specific types of massage [3] and study designs [13]. In accordance with the aforementioned information and the general significance of massage therapy and recovery in sports and exercise, there is a need for further investigations on this topic. Therefore, this review paper aims to search and systemize the current literature findings relating to massages’ effects on sports and exercise performance via motor abilities, neurophysiological, and psychological mechanisms. The main goal of this study is to evaluate the general effects of massage therapy on sports and exercise performances from the relevant studies conducted in the previous four decades, regardless of the study’s methodology, participants, massage techniques, and their modalities or duration times. The results of this review paper could provide important information about the applicability of massage therapy, which could be an important step in future research and the development of the recovery process in sport and exercise. 

## 2. Materials and Methods

This review was written in accordance with the PRISMA (Preferred Reporting Items for Systematic Reviews and Meta-analysis) guidelines [14].

### 2.1. Literature Search Parameters

A search was conducted via PubMed, Cochrane, Web of Science, Scopus, and Google Scholar to strengthen the search strategy’s comprehensiveness. A literature search was conducted in July 2020. The following terms were used in the search: sports massage OR massage exercise OR massage performance OR foam rolling AND muscle force OR muscle strength OR muscle power OR speed OR flexibility OR endurance OR range of motion OR lactate concentration OR blood flow OR temperature OR creatine kinase OR soreness OR electromyography OR anxiety OR mood state OR recovery OR fatigue OR fatigue recovery OR stress.

### 2.2. Eligibility Criteria

Studies were deemed to be eligible if they met the following criteria: they were from the field of massage therapy in sport and exercise and were conducted on human participants. Additionally, they had to be written in English between 1980 and 2020. All study design types were included, except review papers. Additionally, no limitations were set for massage therapy’s type, technique or duration, sport type, physical activity level, and age of the participants. Additionally, massage interventions were implemented in all conditions, before or after the performance, during rest or after fatigue protocol was applied, and over any body region. After the initial search, papers were manually examined to exclude duplicates and irrelevant articles. One hundred and fourteen studies were included in this review. Afterward, the articles were analyzed and systematized, which resulted in seventy-three articles that regarded massages’ effects on motor abilities, forty-eight articles that dealt with its effects on neurophysiological, and fifteen on psychological mechanisms. There were 114 included papers, from which 22 examined different mechanisms in the same study.

## 3. Results

**Figure 1:**
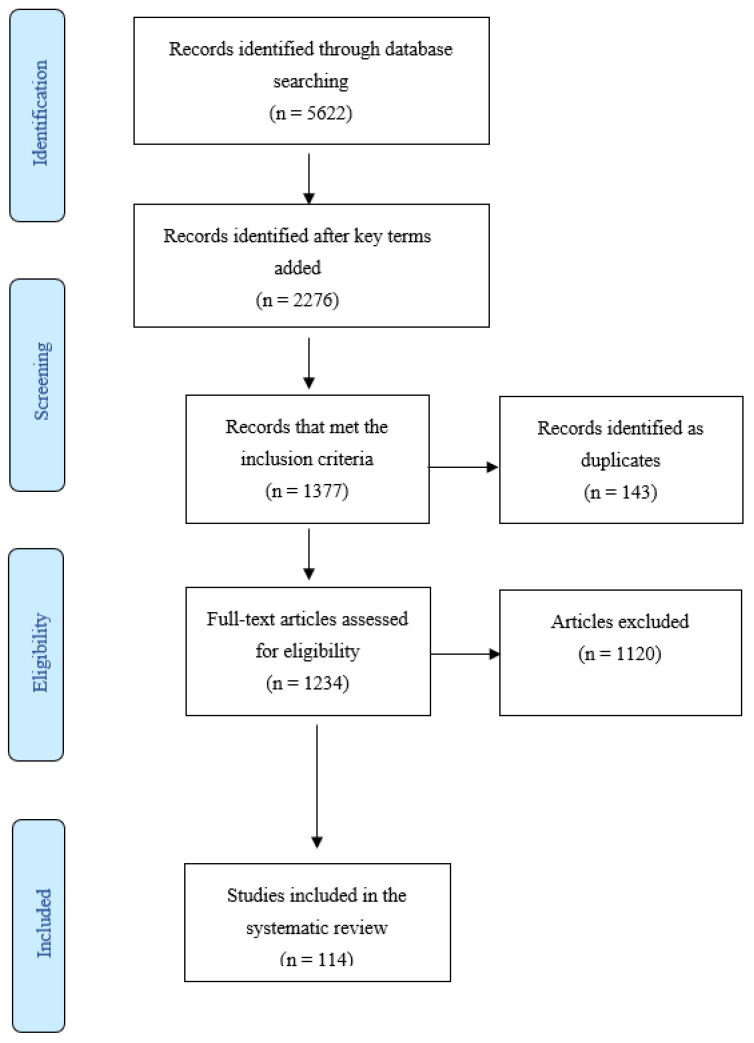
Preferred Reporting Items for Systematic Reviews and Meta-Analysis (PRISMA) flow diagram.

We identified 114 studies that met our eligibility criteria (Figure 1). A total of 2731 participants were included (male = 1596; female = 854; undefined = 281; average age = 26.6 years), substantially exceeding 1012 participants in the largest previous meta-analysis [3]. From a total of 114 studies, in 50 studies, the participants were recreational individuals, 31 studies included athletes, 12 studies included untrained individuals, and 21 studies did not define the subject from the aspect of physical activity. Finally, 66 studies examined the effects of manual massage and 48 examined the effects of different versions of a foam roller, 88 studies examined the effects of massage therapy on leg muscles, 10 examined the effects on arm muscles, and 16 studies examined the effects of massage therapy on different muscle regions.

### 3.1. The Effect of Massage Therapy on Motor Abilities

The effects of massage therapy on motor abilities are presented in Table 1, in which 73 studies were included. Force, strength, speed, endurance, and flexibility are the main motor abilities that were examined in the reported research. 

### 3.2. The Effects of Massage Therapy on Neurophysiological Mechanisms

The effects of massage therapy on neurophysiological mechanisms are presented in Table 2, in which 48 studies were included.

### 3.3. The Effects of Massage Therapy on Psychological Mechanisms

The effects of massage therapy on psychological mechanisms are presented in Table 3, in which 15 studies were included.

## 4. Discussion

This review paper aimed to evaluate massages’ influence on motor abilities and neurophysiological and psychological mechanisms and the main factors contributing to sports and exercise performances. For that purpose, we conducted a comprehensive literature review that included the mentioned factors. To the best of the authors’ knowledge, this review study included the largest number of studies on this topic.

### 4.1. The Effect of Massage Therapy on Motor Abilities

In most studies, massages did not affect muscle force [15,16,17,18,19,20,21,22,23,24,25,26,27,28,29,30,31], but a few studies showed that massages led to a significant improvement in muscle force [32,33,34,35,36,37,38]. They also revealed that performing a massage before doing muscle strength or speed tests in most conditions did not alter the results in the post-tests [18,24,25,27,30,36,41,42,43,44,45,46,47,48,49,50,51,61,62,63,64]. However, the results of several studies showed improvements in muscle force and strength, especially 48 h after the fatigue protocols [21,35,36,47,53,59].

The findings of Hiruma et al. (2014) and Kargarfard et al. (2016) revealed the positive effects of massages on muscle force 48 h after the fatigue protocol [35,36], and McGregor et al. (2018) also obtained positive results of massages on maximal voluntary contraction (MVC), but only 30 min after the treatment. There were no significant improvements when muscle force was immediately measured or 15 min after foam rolling (FR). This was the first study that reported an improvement in MVC following FR alone, suggesting that FR could reduce the impact of fatigue during this submaximal task. The authors concluded that FR allowed the muscle to be activated more efficiently and should be conducted at least 30 min before the activity [39]. Farr et al. (2002) also presented different muscle force results in relation to time after the fatigue protocol. They compared the influence of therapeutic massages on the subjects’ force after a 40 min downhill treadmill walk loaded with 10% of their body mass. The massage was conducted on one limb 2 h post-walk, and muscle force was measured twice before and 1, 24, 72, and 120 h after the walk. The results indicated that the massage negatively affected muscle force only 1 h, but not 24, 72, and 120 h after the walk [40]. Only one study demonstrated that massage therapy negatively affected muscle force development. The findings of that study revealed that a single bout of foam rolling led to neuromuscular exhaustion regarding the maximal force production of knee extensors [41].

When muscle strength was observed, two types of methods were used: strength assessed with a dynamometer [40,41,42,43,44,45,46,52,56,57] and assessed via jumping [18,21,24,25,27,30,33,35,36,40,43,44,45,47,48,49,50,51,53,54,55,58,60]. Most studies indicated that a massage does not affect muscle strength [18,24,25,27,30,36,41,42,43,44,45,46,47,48,49,50,51]. Additionally, most benefits were found 48 h after the massage therapy [21,35,36,47,53], while four studies showed an immediate positive effect of the massage on muscle strength [33,52,54,55]. In the first one, the subjects were given an FR massage and performed a dynamic warm-up exercise [33]. In the second one, the subjects used vibration rolling (VR), non-vibration rolling (NVR), and static stretching as part of their warm-up. Vibration rolling significantly increased the quadriceps’ muscle strength [52]. The third study revealed that both VR and NVR allows the athletes to have increased jump heights [54], and the last one showed that adding VR to dynamic stretching would also result in greater power in the lower limbs [55]. The findings of these studies suggested that using FR as a part of a warm-up should lead to an overall improvement in muscle strength. The opposite results were obtained regarding the strength of the quadriceps and hamstrings muscles when different speeds were used, as measured with an isokinetic dynamometer [40,44,56,57]. In a study by Hunter et al. (2006), massages appeared to reduce strength during concentric isokinetic contractions of the knee extensions at 60°/s, with there being no changes when they were performed at 120, 180, and 240°/s velocities. This reduction was caused by a change in muscle architecture, which affected the length–tension relationship and not due to altered neuromuscular recruitment (44). On the other hand, Arroyo-Morales et al. (2011) showed that the strength of knee extensions was significantly lower when they were performed at higher velocities (180 and 240°/s) and that a massage did not influence them at lower velocities (60 and 120°/s). In this study, a pre-event massage negatively affected muscle performance at higher velocities, possibly because of the increased parasympathetic nervous system activity and decreased afferent input, leading to less motor unit activation. However, the strength of knee flexions stayed unchanged after a massage at all observed velocities [56]. The results of Su et al. (2017) also revealed that a massage could not contribute to knee flexion peak torque improvements. However, that was not the case for the quadriceps muscles. In this study, FR positively affected the knee extension peak torque at 60°/s [57]. As mentioned before, Farr et al. (2002) investigated the influence of a therapeutic massage on hamstrings’ strength and on a single-leg vertical jump height before and 1, 24, 72, and 120 h after 40 min downhill treadmill loaded walking. The subjects performed knee flexions at velocities of 60, 120, and 240°/s. The isokinetic strength at 60°/s and vertical jump height were significantly lower for the massaged leg at 1 and 24 h post-walk. No significant differences were found in the remaining testing variables [40]. Giovanelli et al. (2018) measured the effects of FR on squat jump (SJ) and countermovement jump (CMJ) performances immediately and 3 h after treatment. FR intervention did not modify the maximal lower limbs’ strength during explosive efforts without the storage of elastic energy (SJ), but it improved the maximal power exertion during explosive efforts characterized by the storage of elastic energy (CMJ) [58]. The only study where a massage had a direct negative influence on vertical jumps compared with that of passive rest was found in the research of Arabaci (2008) [60].

Most analyzed studies found that a massage neither accomplished a positive nor a negative effect on sprint performance [48,61,62,63,64], and it did not alter the dynamic reaction time [50]. Arabaci (2008) also examined the effects of a massage on sprint performance. The results revealed that a massage did not change the 20 m flying sprint completion time or the leg reaction time. On the other hand, the massage protocol induced significantly shorter completion times of 10 m and 30 m runs [60].

Concerning endurance, a twenty-minute massage did not improve the cycle ergometer endurance times (total or lap times) during a 161 km race. The race was finished in 4 days, and the same distance had to be completed daily [65]. In a similar endurance study, the subjects did not obtain better results in a 5 km bicycle race when they were given a massage rather than active or passive rest. However, combining massage and active recovery led to better results at the same distance [66]. Junker and Stöggl (2019) also demonstrated that FR did not increase nor decrease core strength endurance within an eight-week training period [51]. The next four studies showed improvements in a leg extension task, where the subjects performed the maximum number of leg extensions against half maximum load [67], in an eggbeater kick performance endurance task (water polo) [68], in a 200 m swimming task m [69], and in a hand grip endurance task involving healthy young men [37].

The most obvious effects of the massage were the effects on flexibility, as most studies indicated a positive correlation between massage and flexibility, significantly increasing the range of motion. Therefore, it is concluded that a massage can be used as an alternative method for the enhancement of flexibility [20,21,24,25,28,29,30,33,34,35,42,46,49,51,54,55,57,60,68,70,71,72,73,74,75,76,77,78,79,80,81,82]. A few studies showed significant and non-significant enhancements in range of motion (ROM) [27,52,83,84]. Aune et al. (2018) showed that FR only led to acute and not chronic improvements of dorsiflexion ROM [27]. Additionally, improvements in hip abduction were found only after FR for the gluteal muscle group and not for the iliotibial band [84]. When vibration and non-vibration rolling were compared, VR affected both knee extension and flexion, and NVR only affected knee extension [52]. The foam rolling of the anterior thigh improved hip extension ROM and did not affect knee flexion ROM [83]. Only six studies showed non-significant enhancements of ROM [16,39,48,85,86,87]. The FR technique was used in all of these studies, and the massage time was no longer than two minutes. The effects of the massage on flexibility 6, 24, and 48 h [16] and 24 h [48] post-exercise were also without significance.

### 4.2. The Effects of Massage Therapy on Neurophysiological Mechanisms

Most studies examining the neurophysiological mechanisms of the human organism and its relationship with massage therapy concerned muscle fatigue or soreness. Fatigue activates recovery mechanisms that protect the organism. The accumulation of lactate acid is one of the most important mechanisms, which leads to the appearance of fatigue. Therefore, its removal is believed to be crucial for recovery [129,130]. Regarding the neurophysiological mechanisms, a massage cannot remove lactate acid, but it can reduce creatine kinase enzyme and, in that way, contribute to reducing pain or delayed onset muscle soreness.

Bale and James (1991) confirmed that a massage has a positive effect on lactate removal (LR) [88]. There was just one more study in which a massage was more effective than passive recovery was in removing blood lactate (10 min massage after 200 m of front crawl swimming with maximal effort) [69]. Nevertheless, one study showed that a massage negatively affected LR [99]. In this study, subjects performed 2 min of strenuous isometric handgrip exercise at 40% MVC to elevate the level of forearm muscle lactic acid after they received a manual massage for 10 min. This was the first study that examined venous lactate acid, allowing researchers to investigate a massage’s influence on its removal from exercised muscle. In a series of future studies, other authors did not obtain similar results [11,12,19,66,87,89,90,91,92,93,94,95].

Researchers tried to explain eventual lactate removal with increased blood flow (BF) and temperature changes during that period. Several methods were used to analyze the relationship between massage and BF; the results differed depending on the method used. Two studies found that massage therapy increased BF. They used a Laser blood flow meter [96], Spectral Doppler, and Power Doppler ultrasound [97]. In the other three studies, the researchers used the Pulsed Doppler method and did not find any effect on BF [15,98] or even impeding factors [99].

Concerning massages’ effects on muscle temperature, Hinds et al. (2004), as well as Mori et al. (2004) and Boguszewski et al. (2014), revealed that a massage has only a surface (skin) influence [96,100,101]. However, Hinds et al. (2004) also measured the temperature of m. vastus lateralis at depths of 3, 2 and 1 cm using a needle thermocouple under local anesthesia. The results of this study did not support the hypothesis that a post-exercise massage elevates limb blood flow [101]. On the other hand, Drust et al. (2003) showed that, besides skin temperature, a massage increased the temperature at 1.5 cm and 2.5 cm and did not have an influence at a depth of 3.5 cm [102]. The experiments showed that blood flow and muscle temperature did not help remove lactate acid.

One of the most common fatigue mechanisms is DOMS. It was believed that lactate acid is the main cause of DOMS. However, Cheung et al. (2003) have proven that the increased lactate acid concentration after exercise returns to rest values one hour after strenuous exercise [131]. Therefore, it can be concluded that higher values of lactate acid cannot cause DOMS, which is formed from 24 to 48 h after intense physical activity. A detailed investigation of DOMS and intense physical activity revealed the development of muscle fibers damage, which led to the direct releasement of the enzyme creatine kinase (cK) [36,103,104,105,106]. Only one study did not show the positive influence of massages on the reduction of the cK level [107]. In this experiment, the massage therapist conducted vigorous massages, increasing the amount of cK. It eventually led to a subject feeling of reduced DOMS. This enzyme is one of the main indicators of damaged muscles, activating pain receptors and elevating their perception. In conclusion, there are positive trends between massage application and the reduction of DOMS, which are backed up by the results of numerous studies: [16,26,38,40,45,48,59,77,108,109,110,111,112].

Regarding muscle activity, it has been assumed that a massage could influence the level of muscle activation. Because of this, researchers tried to investigate those claims. Surface electromyography is a technique used for capturing and measuring electrical activity and muscle action potential. It is commonly applied to specify force production and analyze muscle fatigue. Two methods are used to assess the differences among EMG signals. First, the root means square (RMS) value of the myoelectric signal is a commonly utilized method that reflects the level of the physiological activities in the motor unit during contraction [132]. Most of the studies showed that a massage could not alter the electromyographic characteristics of muscles [34,44,95,113,114,115]. On the other hand, a few studies demonstrated the double-natured effects of massages on the EMG properties of muscles [29,125,126]. In the study of Madoni et al. (2018), the subjects performed maximal knee extension and flexion at three different velocities. No significant changes were found for eccentric hamstring EMG, while the concentric muscle activation of the biceps femoris decreased from pre- to post-test after the foam rolling of the dominant side hamstrings [29]. Aboodarda et al. (2017) investigated the alterations of corticospinal excitability following the rolling massage of the quadriceps muscles. The RMS EMG recorded from VL and VM at 50% of MVC did not demonstrate any difference between the two conditions, but it indicated a significantly lower value for electromyographic activity recorded from VL at 10% MVC. The results revealed that rolling massages aggravate the central excitability of muscles (specifically VL), but only at low-level contractions where the minimum central drive is required to recruit the low threshold spinal motoneurons and motor units [116]. Cavanaugh et al. (2017) examined antagonist muscle activation after FR. They inspected what was happening with quadriceps after biceps femoris rolling and vice versa. The results revealed decreased hamstring activation following the quadriceps foam rolling intervention, but not vice versa. It appeared that FR could alter antagonist muscle activity. The authors attributed this to greater level of perceived pain with quadriceps rolling [117]. Another method, which assessed the differences among EMG signals, used Hoffman reflex (H-reflex) and M-wave measures. Two studies revealed an immediate return of the H-reflex to the baseline after a massage, proving that a massage can induce the modulation of spinal excitability [118,119]. These findings were in line with a study by Sullivan et al. (1991) who found that the H-reflex amplitude was noticeably reduced during the performance of a massage compared with that during the period before or after massage [133]. It was also noticed that excitability, observed through the H-reflex, decreases when the level of applied pressure is higher [119]. It means that a massage can instantly reduce motoneuron activity, and that reduction depends upon the type, duration, and location of the massage.

### 4.3. The Effects of Massage Therapy on Psychological Mechanisms

Studies that link massage and psychological mechanisms are scarce. In the athletic and recreational sport population, most of the studies confirmed a positive correlation between a massage and the improvement of different psychological states. Massages reduce stress [122,124], anxiety [120,121,122,123], depression [122], and fatigue perception [92,93,96,112,125,126,127] and increase mood [56,120,121], relaxation [120] and recovery from fatigue [91,124,125]. However, only one study found no influence of massages on mood state [128]. In that study, 16 subjects completed a questionnaire to establish their baseline mood, and then performed a 30 s Wingate anaerobic cycling test. After the test, they received a 30 min massage or had 30 min of passive rest, and then repeated the same procedure.

## 5. Conclusions

The massage generally does not cause negative or positive effects on motor performance after its application, except flexibility, and there are some indications of its positive effects 48 h after intensive activities. Concerning the neurophysiological parameters, a massage did not affect blood lactate clearance, muscle blood flow, muscle temperature, or activation. However, many studies indicate pain reduction and delayed onset muscle soreness, which are probably correlated with the reduction of the level of creatine kinase enzyme and psychological mechanisms. Nevertheless, massage therapy is often used in modern elite sports and exercise, probably because of its effects on different psychological states, such as decreases in depression, stress, anxiety, and fatigue perception and increases in mood, relaxation, and the perception of recovery.

## Figures and Tables

**Table 1:** Massage therapy effects on motor abilities.

STUDIES	TYPE OF MASSAGE	MOTOR ABILITY	RESULTS
	Man = N (Studies)		
	FR = N (Studies)		
[15,16,17,18,19,20,21,22,23,24,25,26,27,28,29,30,31]	Man = 8 [15,16,17,18,19,22,23,31]	Force [17]	/
	FR = 9 [20,21,24,25,26,27,28,29,30]		
[32,33,34,35,36,37,38]	Man = 4 [32,35,36,37] FR = 3 [33,34,38]	Force [7]	↑
[39]	FR	Force [2]	/(0, 15 min); ↑ (30 min)
[40]	Man		/(24 h, 72 h, 120 h); ↓ (1 h)
[41]	FR	Force [1]	↓
[18,24,25,27,30,36,41,42,43,44,45,46,47,48,49,50,51]	Man = 8 [18,36,42,43,44,45,46,47] FR = 9 [24,25,27,30,41,48,49,50,51]	Strength [17]	/
[21,33,35,36,47,52,53,54,55]	Man = 4 [35,36,47,53] FR = 5 [21,33,52,54,55]	Strength [9]	↑
[40]	Man	Strength [6]	/KF 60°/s (72 h, 120 h); ↓ (1 h, 24 h);/KF 120, 240°/s (1 h, 24 h, 72 h, 120 h);/VJ (72 h, 120 h); ↓ (1 h, 24 h)
[44]	Man		/KE (120, 180, 240°/s); ↓ KE (60°/s);
[56]	Man		/KE (60, 120°/s); ↓ KE (180, 240°/s);/KF (60, 120, 180, 240°/s)
[57]	FR		/KF 60°/s; ↑ KE 60°/s (DS and FR)
[58]	FR		/SJ (Post, 3 h); ↑ CMJ (Post, 3 h)
[59]	FR		/(1 h, 72 h); ↑ (24 h, 48 h) compared with PR
[60]	Man	Strength [1]	↓
[48,50,61,62,63,64]	Man = 3 [61,62,63]	Speed [6]	/
	FR = 3 [48,50,64]		
[60]	Man	Speed [1]	↓ (10 mAcc, 30 mSP); /(FS20mSP, LRT)
[51,65,66]	Man = 2 [65,66]	Endurance [3]	/
	FR = 1 [51]		
[37,67,68,69]	Man = 4	Endurance [4]	↑
[20,21,24,25,28,29,30,33,34,35,42,46,49,51,54,55,57,60,68,70,71,72,73,74,75,76,77,78,79,80,81,82]	Man = 10 [35,42,46,51,54,55,60,68,70,71]	Flexibility [32]	↑
	FR = 22 [20,21,24,25,28,29,30,33,34,49,57,72,73,74,75,76,77,78,79,80,81,82]		
[27]	FR	Flexibility [4]	↑ (Acute); /(Chronic)
[52]	FR		↑ KE; ↑ KF (VR); ↑ KE;/KF (NVR)
[83]	FR		↑ HE (FR of AT); /KF (FR of AT)
[84]	FR		↑ (FR of GMG); /(FR of ITB)
[16,39,48,85,86,87]	Man = 1 [16]	Flexibility [6]	/
	FR = 5 [39,48,85,86,87]		

Legend: Man: Manual massage; FR: Foam rolling; ↑: Increase effect; ↓: Decrease effect; /: Without effect; KE: Knee extension; KF: Knee flexion; HE: Hip extension; DS: Dynamic stretching; SJ: Squat jump; CMJ: Counter movement jump; PR: Passive rest; Acc: Acceleration; SP: Sprint; FS: Flying start; LRT: Leg reaction time; AT: Anterior thigh; GMG: Gluteal muscle group; ITB: Iliotibial band; VR: Vibration rolling; NVR: Non-vibration rolling.

**Table 2:** Massage therapy effects on neurophysiological parameters.

STUDIES	TYPE of MASSAGE	NEUROPHYSIOLOGICAL PARAMETER	RESULTS
	Man = N (Studies)		
	FR = N (Studies)		
[69,88]	Man = 2	Lactate Removal [2]	↑
[19,66,87,89,90,91,92,93,94,95]	Man = 9 [19,66,89,90,91,92,93,94,95]	Lactate Removal [10]	/
	FR = 1 [87]		
[88]	Man	Lactate Removal [1]	↓
[96,97]	Man = 1 [96]	Blood Flow [2]	↑
	FR = 1 [97]		
[15,98]	Man = 1 [15]	Blood Flow [2]	/
	FR = 1 [98]		
[99]	Man	Blood Flow [1]	↓
[96,100]	Man = 2	Temperature [2]	↑
[101]	Man	Temperature [2]	↑ (ST); /(MT 1, 2, 3 cm)
[102]	Man		↑ (ST, MT 1.5, 2.5 cm); /(MT 3.5 cm)
[36,103,104,105,106]	Man = 5	Creatine Kinase [5]	↓
[107]	Man	Creatine Kinase [1]	↑
[16,26,38,40,45,48,59,77,108,109,110,111,112]	Man = 7 [16,40,45,108,109,111,112]	DOMS [13]	↓ (Trend)
	FR = 6 [26,38,48,59,77,110]		
[34,44,95,113,114,115]	Man = 4 [44,95,113,114]	EMG RMS [6]	/
	FR = 2 [34,115]		
[29]	FR	EMG RMS [3]	/ECC HM; ↓ CON HM
[116]	FR		/50% MVC; ↓ 10% MVC
[117]	FR		/QC after HM FR, ↓ BF after QC FR
[118,119]	Man = 1 [119]	EMG H-reflex, M-wave [2]	↓ H-reflex;/M-wave
	FR = 1 [118]		

Legend: Man: Manual massage; FR: Foam rolling; DOMS: Delayed onset muscle soreness; ↑: Increase effect; ↓: Decrease effect; /: Without effect; EMG: Myoelectrical activity; RMS: Root mean square; ST: Surface temperature; MT: Muscle temperature at different depths; ECC: Eccentric EMG activation; CON: Concentric EMG activation; QC: Quadriceps; HM: Hamstrings; BF: Biceps femoris.

**Table 3:** Massage therapy effects on psychological parameters.

STUDIES	TYPE of MASSAGE	PSYCHOLOGICAL PARAMETER	RESULTS
	Man = N (Studies)		
	FR = N (Studies)		
[120,121,122,123]	Man = 4	Anxiety [4]	↓
[122,124]	Man = 2	Stress [2]	↓
[122]	Man	Depression [1]	↓
[125]	Man	Self-reported perceptions of physical symptoms [1]	↓
[92,93,96,112,125,126,127]	Man = 6 [92,93,96,112,125,127]	Fatigue perception [7]	↓
	FR = 1 [126]		
[91,124]	Man = 2	Perception of recovery [2]	↑
[56,120,121]	Man = 2 [120,121]	Mood state [3]	↑
	FR = 1 [56]		
[128]	Man	Mood state [1]	/
[120]	Man	Relaxation [1]	↑
[125]	Man	Positive affect [1]	↑

Legend: Man: Manual massage; FR: Foam rolling; ↑: Increase effect; ↓: Decrease effect; /: Without effect.

## Data Availability

All data are available in the manuscript.
